# Positive Behavioral, Morphophysiological, and Gene Expression Effects of the Administration of Virgin Coconut Oil in an Ischemic Stroke Surgical Rat Model

**DOI:** 10.3390/ijms26136215

**Published:** 2025-06-27

**Authors:** Rodel Jonathan S. Vitor, Ryota Tochinai, Shin-Ichi Sekizawa, Masayoshi Kuwahara

**Affiliations:** 1 Laboratory of Veterinary Pathophysiology and Animal Health, Department of Veterinary Medical Sciences, Graduate School of Agricultural and Life Sciences, The University of Tokyo, Tokyo 113-8657, Japan; rsvitor@up.edu.ph (R.J.S.V.II); ssekizaw@g.ecc.u-tokyo.ac.jp (S.-I.S.); 2 Department of Biology, College of Arts and Sciences, University of the Philippines Manila, Manila 1000, Philippines

**Keywords:** gene expression, neuronal damage, stroke, surgical model, virgin coconut oil

## Abstract

Stroke is still considered a predominant cause of morbidity and mortality, for which research on prevention and cure has been sought to prevent neuronal damage after a stroke incident. In this research, we evaluated the protective effects of virgin coconut oil (VCO) using behavioral, morphophysiological, and gene expression parameters using an ischemic stroke surgical rat model using Sprague Dawley (SD) rats. Eight-week-old SD rats were subjected to repeated oral administration (5 mL/kg/day) of either 1% Tween 80 or VCO. For behavioral and morphophysiological parameters, surgery was performed for each group, after which neurological scoring was performed at 4 h, 24 h, 48 h, 5 d, and 10 d. Further, hematological and brain morphology assessment was performed after euthanasia and necropsy of the animals. For gene expression studies, surgery was performed with animals sacrificed at different time points (baseline, before surgery, 4 h, 24 h, and 48 h after surgery) to collect the brain. Results of the study showed that there are differences in the neurological scores between the two treatments 24 h, 48 h, and 5 d after surgery. Brain morphology assessment also showed favorable results for VCO for infarct size, edema, and hypoxic neurons. Gene expression studies also showed positive results with an increase in the relative expression of angiogenin (*Ang*), angiopoietin (*Angpt 1*), *Parkin*, dynamin-related protein 1 (*Drp 1*), mitofusin 2 (*Mfn 2*), and mitochondrial rho (*Miro*) and decreased relative expression of caspase 3, receptor for advanced glycation end-product *(Rage*), and glyceraldehyde-3-phosphate dehydrogenase (*Gapdh*). In summary, the current study shows that VCO may have protective effects on the brain after stroke, which may be explained by the results of the gene expression studies.

## 1. Introduction

In the clinical practice of stroke management in humans, physiological variables such as blood pressure (BP), heart rate (HR), temperature, and blood glucose have been routinely monitored, as they have been found to affect the outcomes of stroke [1]. Similarly, hematological changes [2] and behavioral studies, conducted to mimic dysfunctions in humans [3], are performed in experimental stroke studies in rats and other laboratory animals to determine neurologic improvement. Neurologic deficits post-stroke often led to permanent body function impairments, disabilities, and a compromised lifestyle [4,5,6,7,8]. Motor impairments are the most prevalent and documented neurologic deficit observed post-stroke in human and animal studies, with partial to total loss of cranial nerve function, muscle power, muscle tone, balance, coordination, gait, reflexes, and apraxia included in this domain [9]. These clinical signs are assessed regularly in stroke patients and those in rehabilitation to evaluate their recovery. They are also used for developing neurologic examination scores for stroke models in different animals [10]. Further, brain analysis for infarction, edema, and hypoxic neurons is likewise taken into consideration in most animal studies [11,12].

**Figure 1:**
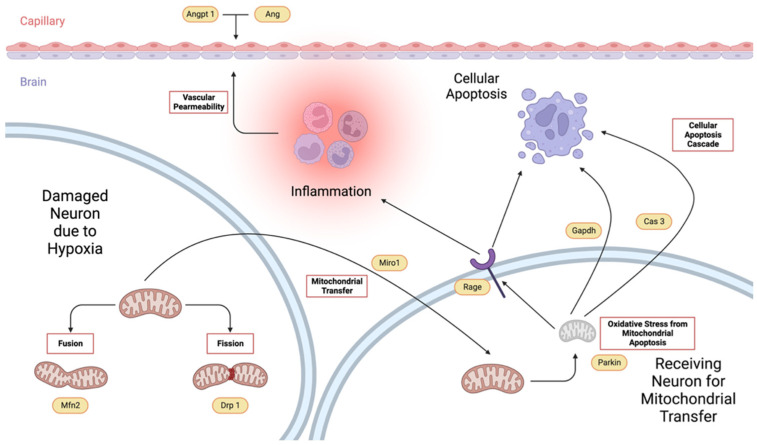
Schematic diagram showing the interrelationship of the different genes associated with cellular apoptosis, mitochondrial dynamics, and blood vessel permeability. (Created with BioRender.com).

Our previous study [13] showed that virgin coconut oil (VCO) may delay the incidence of stroke and prolong the survival of animals. However, given the limitations of the stroke-prone spontaneously hypertensive rat (SHRSP) model, since the timing of stroke incidence is not similar within the different animals, a surgical model is preferred to determine parameters that can be affected by the time after a stroke occurs. Further, understanding the mechanism of the therapeutic effects of VCO in stroke is warranted to further assist in unraveling its mechanism of action, which may support the use of VCO in translational and clinical research. Thus, molecular markers found in the brain are warranted to elucidate the possible mechanism of VCO therapeutic effect. In this study, the following genes will be focused on: caspase-3 (*Cas 3*), receptor for advanced glycation end-products (*Rage*), angiogenin (*Ang*), angiopoietin 1 (*Angpt 1*), dynamin-related protein 1 (*Drp 1*), mitofusin 2 (*Mfn 2*), parkin, mitochondrial rho (*Miro 1*), and glyceraldehyde-3-phosphate dehydrogenase (*Gapdh*), with hypoxanthine phosphoribosyl transferase 1 (*Hprt 1*) used as an internal control (Figure 1).

It was established that VCO may confer favorable effects in an SHRSP model by attenuating hypertension and possibly conferring neuroprotective effects in the brain. Thus, this study aims to determine the effects of VCO in a surgical model of ischemic stroke in Sprague Dawley (SD) rats, specifically on neurological behavior scores, hematological parameters, and histomorphology of the brain. Further, this study aims to elucidate the possible mechanisms of neuroprotection by using a surgical model of ischemic stroke only within the acute phase of the stroke phenomena.

## 2. Results

### 2.1. Results of the Esterification/Gas Chromatography Analysis of VCO

Based on the analysis conducted using the esterification/gas chromatography method, it was observed that the predominant fatty acids in the sample used are lauric acid, myristic acid, and palmitic acid (Table 1).

### 2.2. Neurologic Scores

**Figure 2:**
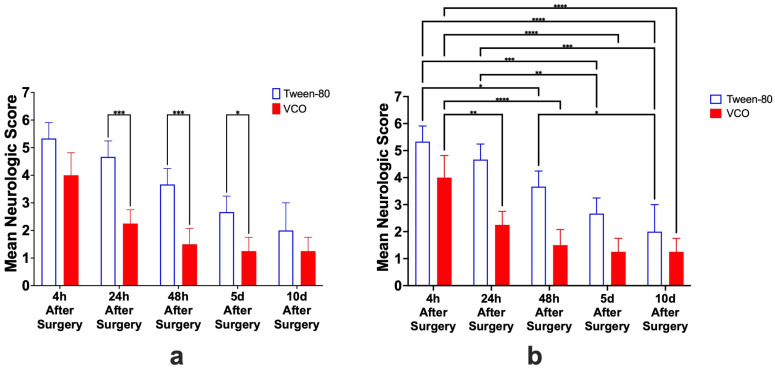
The mean neurologic scores (mean ± SD) of SD rats administered with either Tween-80 or virgin coconut oil (VCO) showing the effects of treatment within time points (**a**) and the effects of time on the treatment administered (**b**) (*n =* 5 per group per time point). * *p* < 0.05, ** *p* < 0.01, *** *p* < 0.001, **** *p* < 0.0001 (Bonferroni’s test).

It was observed that the neurologic scores at different time points were decreasing from the start of observation after surgery until euthanasia on day 10. Statistically, it was determined that the interaction of time and the treatment administered were not different (*p* = 0.1317). However, the effect of time (*p* < 0.0001) and the effect of treatment administration (*p* < 0.0001) alone were significant. Specifically, the Tween-80 group and VCO group were found to be not different at 4 h (*p* = 0.0519) and 10 d (*p* = 0.6576), while 24 h (*p* = 0.0002), 48 h (*p* = 0.0007), and 5 d (*p* = 0.0344) after surgery were found to be significant (Figure 2a). Among the different time points within the Tween-80, the following were found to be not significant: 4 h and 24 h after surgery (*p* ≥ 0.9999), 24 h and 48 h after surgery (*p* = 0.6315), 48 h and 5 d after surgery (*p* = 0.6315), and 5 d and 10 d after surgery (*p* ≥ 0.9999); while the following comparisons were significant: 4 h and 48 h after surgery (*p* = 0.0336), 4 h and 5 d after surgery (*p* = 0.002), 4 h and 10 d after surgery (*p* ≤ 0.0001), 24 h and 5 d after surgery (*p* = 0.0066), 24 h and 10 d after surgery (*p* = 0.0002), and 48 h and 10 d after surgery (0.336). On the other hand, among the different time points within the VCO group, the following were found to be not significant: 24 h and 48 h after surgery (*p* ≥ 0.9999), 24 h and 5 d after surgery (*p* = 0.3383), 24 h and 10 d after surgery (*p* = 0.3383), 48 h and 5 d after surgery (*p* ≥ 0.9999), 48 h and 10 d after surgery (*p* ≥ 0.9999), and 5 d and 10 d after surgery (*p* ≥ 0.9999); while the following comparisons were significant: 4 h and 24 h after surgery (*p* = 0.0059), 4 h and 48 h after surgery (*p* ≤ 0.0001), 4 h and 5 d after surgery (*p* ≤ 0.0001), and 4 h and 10 d after surgery (*p* ≤ 0.0001) (Figure 2b).

### 2.3. Hematological Parameters

**Figure 3:**
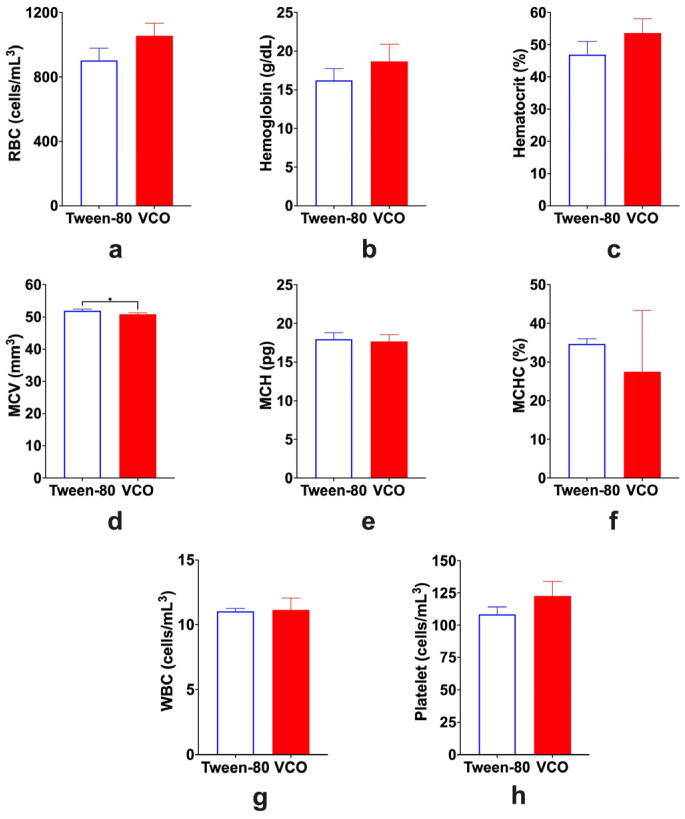
The total red blood cell count (RBC) (**a**), hemoglobin (**b**), hematocrit (**c**), mean corpuscular volume (MCV) (**d**), mean corpuscular hemoglobin (MCH) (**e**), mean corpuscular hemoglobin concentration (MCHC) (**f**), total white blood cell count (WBC) (**g**), and total platelet count (**h**) (mean ± SD) of SD rats administered with either Tween-80 or virgin coconut oil (VCO) (*n =* 5 per group). * *p* < 0.05 (*t*-test).

Among the different hematological parameters analyzed, only the MCV (mean corpuscular volume) (*p* = 0.0369) was significantly different between the Tween-80 group and the VCO group, while the total red blood cells (RBC) (*p* = 0.0508), hemoglobin (*p* = 0.1635), hematocrit (*p* = 0.0983), mean corpuscular hemoglobin (MCH) (*p* = 0.6729), mean corpuscular hemoglobin concentration (MCHC) (*p* = 0.4807), total white blood cells (WBC) (*p* = 0.8411), and total platelet (*p* = 0.1103) were not significantly different from each other (Figure 3).

### 2.4. Infarct Size and Brain Edema

**Figure 4:**
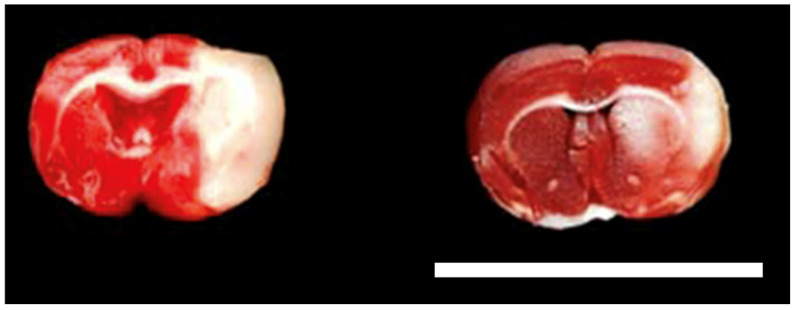
Coronal cut of representative sections of the cerebral area at 2 mm after the level of the bregma of SD rats administered with either Tween-80 (**left**) or virgin coconut oil (VCO) (**right**), showing infarcted area (pale staining region) at day 10. Bar scale—10 mm.

**Figure 5:**
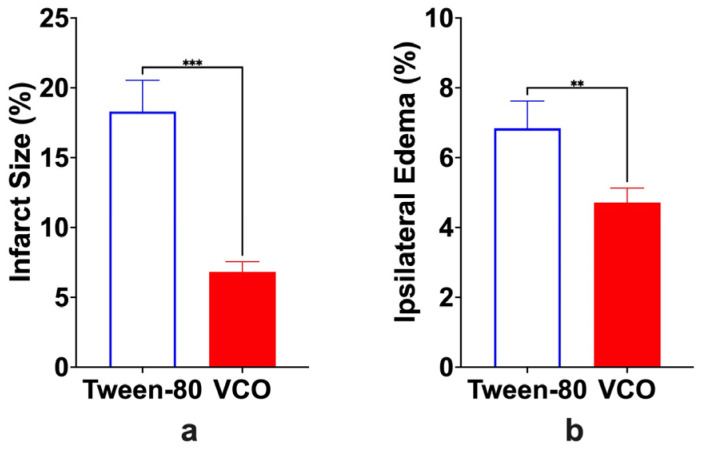
The infarct size volume (**a**) and ipsilateral hemispheric edema (**b**) (mean ± SD) of SD rats administered with either Tween-80 or virgin coconut oil (VCO) at day 10 (*n =* 5 per group). ** *p* < 0.01, *** *p* < 0.001 (*t*-test).

It was observed that infarct size is lesser in size in the VCO group than in the Tween-80 group (Figure 4). Specifically, it was determined that the difference between the two groups was significant, with approximately a 12% difference (*p* = 0.0002). Edema of the ipsilateral hemisphere was also found to be significant (*p* = 0.0053) (Figure 5).

### 2.5. Results of the Histopathological Analysis of Brain Tissues

**Figure 6:**
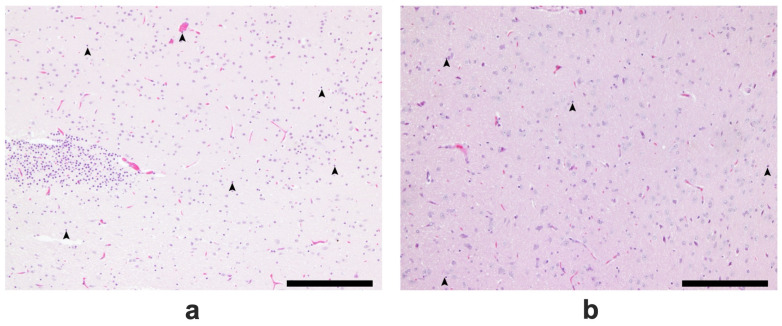
H&E staining of a brain section showing the neocortex of SD rats administered with Tween-80 (**a**) and virgin coconut oil (VCO) (**b**). Legend: arrowhead—leukocyte infiltrates. Bar scale—200 μm.

**Figure 7:**
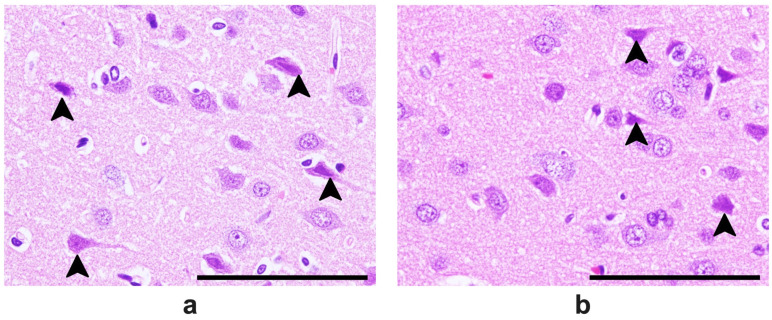
H&E staining of a brain section showing the neocortex of SD rats administered with Tween-80 (**a**) and virgin coconut oil (VCO) (**b**). Legend: arrowhead—hypoxic neurons. Bar scale—200 μm.

**Figure 8:**
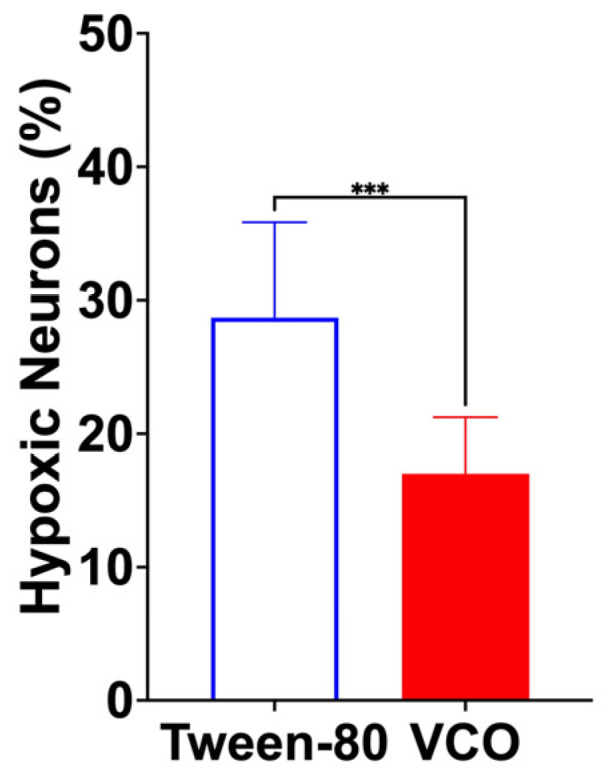
The hypoxic neurons in percentage (mean ± SD) of SD rats administered with either Tween-80 or virgin coconut oil (VCO) (*n =* 5 per group). *** *p* < 0.001 (*t*-test).

At lower magnification, more infiltrative leukocytes are present in the Tween-80-treated group than in the VCO group (Figure 6). At higher magnification, more hypoxic neurons were found in the group treated with Tween-80 (average of 23/80.2 cells) than in the VCO group (average of 13.4/78.8 cells) (*p* = 0.0004) (Figure 7 and Figure 8).

### 2.6. Gene Expression

All the relative gene expressions of *Ang*, *Angpt 1*, *Parkin*, *Drp 1*, *Mfn 2*, *Miro*, *caspase 3*, *Rage*, and *Gapdh* were found to have significant interaction effects (*p* < 0.0001). Testing for individual effects of treatment and time showed that the relative gene expression levels of *Ang*, *Angpt 1*, *Parkin*, *Drp 1*, *Mfn 2*, and *Miro* were higher in the VCO group as compared with the control group. On the other hand, the relative gene expression levels of *caspase 3*, *Rage*, and *Gapdh* were found to be lower in the VCO group as compared with the VCO group.

#### 2.6.1. Effects of Treatment Administration Within Different Time Points

**Figure 9:**
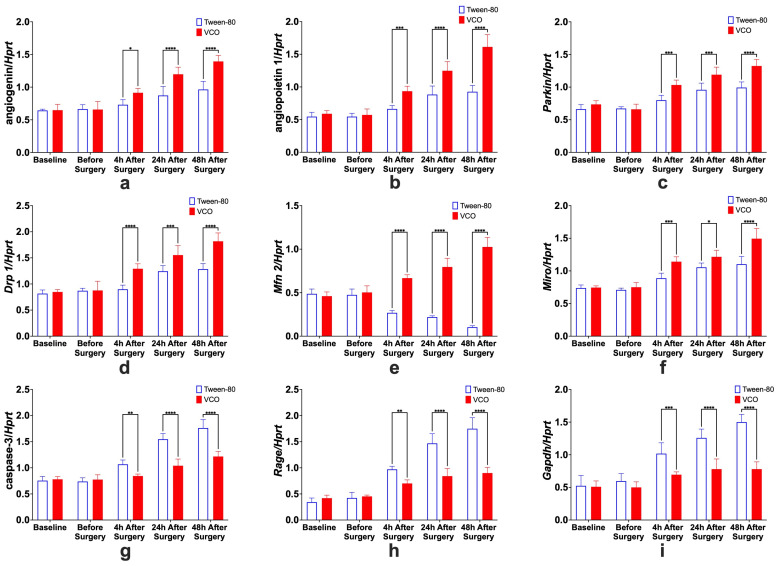
Relative gene expression of angiogenin (**a**), angiopoietin 1 (**b**), parkin (**c**), dynamin-related protein 1 (*Drp 1*) (**d**), mitofusin 2 (*Mfn 2*) (**e**), mitochondrial rho (*Miro*) (**f**), caspase-3 (**g**), receptor for advanced glycation end-products (*Rage*) (**h**), and glyceraldehyde-3-phosphate dehydrogenase (*Gapdh*) (**i**) (mean ± SD) in the left cerebral cortex of Sprague Dawley (SD) rats administered with either Tween-80 or virgin coconut oil (VCO), showing the effects of treatment at different time points. Legend: *Hprt*—hypoxanthine phosphoribosyl transferase 1 (*n =* 5 per group per time point). * *p* < 0.05, ** *p* < 0.01, *** *p* < 0.001, **** *p* < 0.0001 (Bonferroni’s test).

It was observed that the relative expression of *Ang*, *Angpt 1*, *Parkin*, *Drp 1*, *Mfn 2*, *Miro*, *caspase 3*, *Rage*, and *Gapdh*, in the Tween-80 group and the VCO group are not significantly different at baseline and before surgery. In contrast, significant differences are observed at 4 h, 24 h, and 48 h after surgery (Figure 9). This could further strengthen the idea that the ECICAO model may be used as an alternative to the MCAO model, which produces more mortality than the current model used.

#### 2.6.2. Effects of Different Time Points Within Treatment Groups

**Figure 10:**
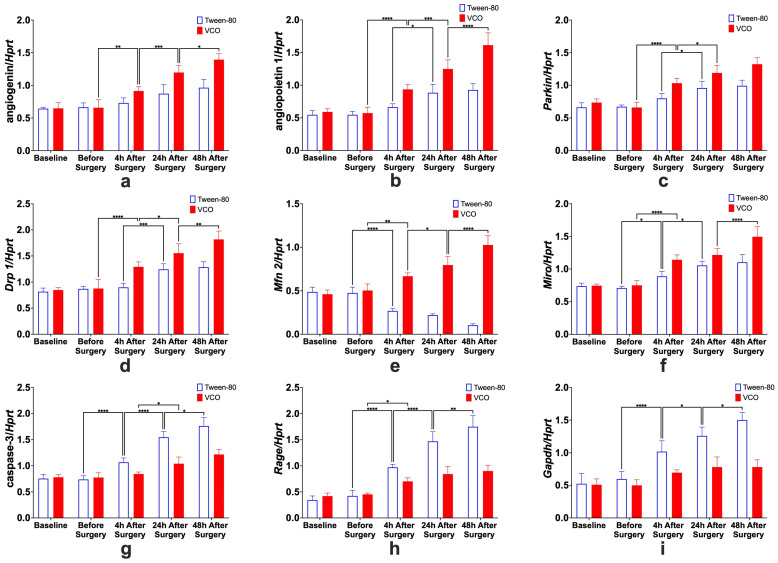
Relative gene expression of angiogenin (**a**), angiopoietin 1 (**b**), parkin (**c**), dynamin-related protein 1 (*Drp 1*) (**d**), mitofusin 2 (*Mfn 2*) (**e**), mitochondrial rho (*Miro*) (**f**), caspase-3 (**g**), receptor for advanced glycation end-products (*Rage*) (**h**), and glyceraldehyde-3-phosphate dehydrogenase (*Gapdh*) (**i**) (mean ± SD) in the left cerebral cortex of Sprague Dawley (SD) rats administered with either Tween-80 or virgin coconut oil (VCO), showing the effects of time on the treatment administered. (*n =* 5 per group per time point). Legend: *Hprt*—hypoxanthine phosphoribosyl transferase 1. * *p* < 0.05, ** *p* < 0.01, *** *p* < 0.001, **** *p* < 0.0001 (Bonferroni’s test).

Increased relative expression of *Ang*, *Angpt 1*, *Parkin*, *Drp 1*, *Mfn 2*, *and Miro* and decreased relative expression of cleaved *Cas 3*, *Rage*, *and Gapdh* were observed in the VCO group across different time points (Figure 10).

1. Angiogenin. For the specific comparison of *Ang* relative gene expression levels within groups, significant differences between before surgery and 4 h after surgery, 4 h and 24 h after surgery, and 24 h and 48 h after surgery, only for the VCO group.
2. Angiopoietin. The effects of time within groups for *Angpt 1* show significant differences between 4 h and 24 h after surgery for the Tween-80 group and between before surgery and 4 h after surgery, 4 h and 24 h after surgery, and 24 h and 48 h after surgery for the VCO group.
3. Parkin. Relative gene expression of parkin between different time points within groups shows that 4 h and 24 h after surgery are different for the Tween-80 group, while before surgery and 4 h, and 4 h and 24 h after surgery are different for the VCO group.
4. Dynamin-Related Protein 1. The relative gene expression of *Drp 1* within groups across time shows that the Tween-80 group is only significant between 4 h and 24 h after surgery. In comparison, before surgery and 4 h after surgery, 4 h and 24 h, and 24 h and 48 h after surgery are significant from each other for the VCO group.
5. Mitofusin 2. For *Mfn 2*, gene expression for the Tween-80 group across time is only significant before and 4 h after surgery; while before surgery and 4 h after surgery, 4 h and 24 h after surgery, and 24 h and 48 h after surgery are different for the VCO group.
6. Mitochondrial Rho. Similar observations for mitofusin 2 are observed in the relative gene expression of *Miro* across time points.
7. Caspase 3. Relative expression of caspase 3 shows differences between before surgery and 4 h after surgery, 4 h and 24 h after surgery, and 24 h and 48 h after surgery for the Tween-80 group, while the VCO group is only different between 4 h and 24 h after surgery.
8. Receptor for Advanced Glycation End-Products. For the relative expression of the receptor for advanced glycation end-products, the Tween-80 group shows differences between before surgery and 4 h after surgery, 4 h and 24 h after surgery, and 24 h and 48 h after surgery, while the VCO group is only different before surgery and 4 h after surgery.
9. Glyceraldehyde-3-Phosphate Dehydrogenase. Though glyceraldehyde-3-phosphate dehydrogenase has been commonly used as an internal control, it has been shown that its expression is affected in ischemic models. Specifically, it was observed that the Tween-80 group is different before surgery and 4 h after surgery, 4 h and 24 h, and 24 h and 48 h after surgery for the Tween-80 group.

## 3. Discussion

The gas chromatography analysis showed that the samples used for the experiment are similar to other virgin coconut oils that have been used in other studies [14]. Numerous studies have demonstrated a consistent fatty acid profile in virgin coconut oil, predominantly composed of medium-chain triglycerides, including lauric acid and myristic acid. This consistency underscores the oil’s stability and purity across various research samples. The repeated findings affirm the health benefits and nutritional value attributed to virgin coconut oil.

Results of the neurologic score show that the animals treated with VCO are different from the control. Since behavior has been attributed to the size of the infarct and/or damage caused by stroke induction [15], it can be said that virgin coconut oil was able to positively affect the behavior post-stroke induction. The functional outcome of stroke assault has been increasingly recognized in preclinical studies to provide therapeutic significance [16]. The most common clinical manifestation of brain damage is motor impairment exhibited by patients who suffered from stroke and animal models induced with cerebral ischemia. Different authors have modified the neurological deficit-scoring test to suit their study, and the most commonly used modification is performing a simple neurological examination evaluating forelimb flexion, resistance to lateral push, and circling behavior [9]. However, more sophisticated assessments such as those performed by Wahl et al. [17] provide a more detailed description of ischemia-induced sensorimotor dysfunctions.

There is a lack of research analyzing hematological parameters in stroke research in rats. Among those studied, much has been focused on the hematocrit [1] as well as on the total red blood cell and white blood cell count [2]. Since it was indicated that hematological parameters are associated with the prognosis of stroke [18], they were analyzed in the current study. However, given the limited references that can be attributed to the hematological changes in the current study, we can only speculate based on current human studies [18,19]. In the study, it was observed that the total red blood cell count, hemoglobin, hematocrit, total white blood cell count, and total platelet count were higher in the VCO-treated group as compared with the Tween-80 group. On the other hand, the secondary red blood cell indices are higher in the Tween-80 group than in the VCO-treated group. However, among these, only the mean corpuscular volume differs between the two groups. It has been hypothesized that a higher MCV may limit the amount of RBC reaching the brain [20], which may likewise lead to neurological dysfunction. However, given that the hematological values analyzed in this study were only determined 10 days after surgery, derangement in hematology may be more evident in the acute phase of the stroke phenomenon.

When trying to understand the pathophysiology of neurological dysfunction brought about by either traumatic brain injury, stroke, or cerebral ischemia, it is best to correlate it with cellular processes involving glutamate excitotoxicity, calcium overload within the cell, mitochondrial dysfunction, and production of reactive oxygen species leading to either apoptosis or necrosis [21]. Cerebral ischemia usually leads to neuronal damage that is linked through complex pathophysiological mechanisms [22] that may be usually seen in classical neurodegenerative disorders. Among these, the easiest to observe is apoptosis, measured grossly and histopathologically. Triphenyl tetrazolium chloride (TTC) staining is a method to determine which cells are unable to take up the stain and are considered dead [23]. In brief, the TTC salt accepts a proton from the succinate dehydrogenase in the inner membrane of the mitochondria, thus reducing it to formazan, which is red in its insoluble form. Thus, a cell that is inactive or dead will not stain and will appear pale or white [24]. It has also been shown that there are no differences when using the same section used for TTC staining for routine hematoxylin and eosin staining [12]; as such, the protocol was used in this study.

Angiogenin and angiopoietin-1 have similar functions in promoting vascularization in the ischemic penumbra. Moreover, angiopoietin-1 is a protein with various functions but has been shown to be active in providing protective effects to blood vessels, preventing plasma leakage, decreasing or inhibiting vascular inflammation, and preventing endothelial death [25]. It has been determined that the levels of plasma angiogenin and angiopoietin after an incidence of ischemic stroke are low, and it has been attributed to poor outcomes, and interventions that may upregulate its expression can potentially improve stroke outcomes [26]. The elevated transcript levels of angiogenin and angiopoietin-1 observed after surgery imply that administering virgin coconut oil could yield neuroprotective benefits post-stroke.

Maintenance of mitochondrial homeostasis during an ischemic insult has been one of the newer approaches to finding stroke treatment. In this study, it was shown that there was an increase of mitofission- and mitofusion-related genes as well as parkin, which is related to mitophagy. It was shown in previous studies that activating mitochondrial fission can allow the dysregulated mitochondria to be segregated and be taken up by autophagosomes, while fusion of surviving mitochondria can lead to the prevention of rapid degradation of mitochondria, which can ultimately lead to autophagy cascade mechanisms [27]. Further, mitochondrial rho-assisted cellular transfer of mitochondria may provide a protective mechanism by transferring viable mitochondria to nearby cells [28]. These dynamics in the mitochondrial function may be useful, especially in maintaining the high energy demand of the brain and preventing further neuronal apoptosis.

Receptor for advanced glycation end-products (*Rage*) was seen to have a decreased expression in VCO-treated rats after surgery. *Rage* has been shown to produce effects in cellular processes, including apoptosis, autophagy, inflammation, migration, proliferation, and its dysfunction. *Rage* and its ligands may lead to the pathogenesis of numerous human diseases after ligand binding [29,30]. It has been shown that decreased levels of RAGE have been shown to favor inflammation [31], with apoptosis being shown to be decreased in RAGE-deficient cells [32]. Since apoptosis is one of the fundamental pathophysiologic mechanisms in strokes, decreasing apoptosis via downregulation of RAGE may be an important pathway and can be used for further therapeutic considerations in stroke. Caspase-3 expression levels of the VCO-treated rats were seen to be decreased when compared with the negative control after surgery. Caspases mediate programmed cell death, or apoptosis, and proceed to activate and catalyze specific cleavages of many key cellular proteins [33]. As such, it can be said that drugs that target cleavage of caspase-3 should be specific to cellular processes since they can either promote apoptosis or delay it. Decreased relative expression of cleaved *Cas-3*, *Rage*, and *Gapdh* may be correlated to better functional recovery after the stroke incident. Furthermore, since the previous genes have been targeted for neuroprotection, the lowered values may also correlate with neuroprotection compared to the Tween-80 group.

In general, just within minutes after a stroke incident, brain tissue devoid of blood flow is fatally injured and subsequently undergoes apoptosis [34]. A zone of less damaged tissues that is transiently silent due to decreased blood flow but is considered metabolically active then surrounds the core [35]. This region, more commonly known as the ischemic penumbra, represents a region where post-stroke salvage may be possible [36]. In recent studies, it has been shown that apoptosis within the ischemic penumbra may occur after several hours to days [22]. Neuronal apoptosis is an important contributing factor to neurological diseases and has been researched comprehensively [35]. In previous studies, downregulation of proteins that inhibit cellular processes such as apoptosis and inflammation decreases the size of the ischemic penumbra in the brain [36]. In another study, neamine has been shown to significantly decrease caspase-3 and the size of the ischemic penumbra [22]. One of the essential pathways in studying apoptosis as a sequela of ischemic stroke is understanding the caspase-3 pathway [34]. In a study in humans after an ischemic stroke, caspase-3 was also associated with poorer neurological outcomes, whether short-term or long-term, and was seen to increase with larger infarct size [37].

The current study also determined that the number of hypoxic neurons is significantly different in the animals treated with VCO than in the Tween-80 group. This may further support the hypothesis that VCO may provide neuroprotection, together with the different results of the parameters determined in this study as well as in our previous study [13]. It may thus be speculated that the antioxidant effects of VCO [38] may be the reason for decreasing the pathologic effects of hypoxic neurons that may ultimately lead to apoptosis of neurons. The gene expression that was studied in this research showed that neuroprotective effects are seen with decreasing levels of gene expression, which can be attributed to attenuating apoptosis and decreasing the size of the ischemic penumbra as well as histologic damage. These effects have been seen in the current study, where the expression levels of cleaved *Cas 3*, *Rage*, and *Gapdh* decreased while the levels of *Ang*, *Angpt 1*, *Parkin*, *Drp 1*, *Mfn 2*, and *Miro* increased. The changes in the relative expression of such genes may have led, as in previous studies, to the decrease in apoptosis and other sequelae of stroke, which may have led to a decrease in histologic damage, which was observed in the first part of this study as well as in the previous SHRSP model. Thus, with the administration of virgin coconut oil, a decrease in histological damage may be attributed to its effect on the differential gene expression of stroke markers. To further strengthen the position of VCO as a possible adjunct therapeutic management for stroke, understanding its effects on classical mediators of inflammation, such as interleukin-1b (IL-1b), interleukin-6 (IL-6), and tumor necrosis factor α (TNFα), should be studied together with studying specific pathways for neurodegeneration and angiogenesis.

## 4. Materials and Methods

### 4.1. Ethics

All the experiments using rats were conducted in accordance with the Animal Experimentation Guidelines of the University of Tokyo and were approved by the Institutional Animal Care and Use Committee of the Graduate School of Agricultural and Life Sciences at the University of Tokyo (P19-063H02; approved on 27 August 2019).

### 4.2. Animal Rearing Conditions

Eight-week-old Sprague Dawley (SD) rats (Charles River Laboratories Japan, Inc., Kanagawa, Japan) were kept at between 20 and 22 °C with a humidity of 74 ± 2 and on a 12 h light:12 h dark cycle. All the cages, feeders, and water bottles were cleaned and disinfected at least once a week. All the animals were acclimatized for one week before the start of the experimentation.

### 4.3. Esterification/Gas Chromatography Analysis of VCO

Virgin coconut oil purchased from a local supermarket in the Philippines (ProSource Virgin Coconut Oil, ProSource International, Inc., San Juan City, Metro Manila, Philippines) was sent to the Philippine Institute of Pure and Applied Chemistry for fatty acid profiling using the esterification/gas chromatography method.

### 4.4. Behavioral and Morphophysiological Studies

#### 4.4.1. Animal Surgical Procedures

SD rats were randomly assigned into two groups with 25 animals each: 1% Tween-80, chosen to mimic the viscosity of the VCO [39], and VCO, both treatments given orally at 5 mL/kg/day. After one week of acclimatization, treatments were administered. After five days of treatment, the rats were subjected to left extracranial internal carotid artery occlusion (ECICAO) before administering the dose for the 6th day. The procedure performed followed what has been discussed in the paper of Diestro, Omar, Climacosa, Mondia, Arbis, Collantes, Khu, Roxas, and Estacio [11], except that the occlusion was permanent in this model under isoflurane anesthesia. In brief, rats were anesthetized and placed in dorsal recumbency. The neck area was shaved and disinfected with alcohol and povidone-iodine. A two-cm midline skin incision was made at the ventral side of the neck region. Subcutaneous tissues, mandibular salivary glands, and the sternohyoid muscles were deflected laterally to expose the left internal carotid artery. The left internal carotid artery was carefully dissected free from surrounding tissues and was ligated using a 3-0 surgical filament. The surgical site was observed for bleeding and hemorrhage, after which the incision site was sutured with 3-0 absorbable suture material. The sutured site was disinfected with povidone-iodine. Animals were then sacrificed under isoflurane anesthesia on the 10th day after surgery.

#### 4.4.2. Neurologic Scoring

The neurologic scoring developed by Bederson, Pitts, Tsuji, Nishimura, Davis, and Bartkowski [9] was used to identify deficits at 4 h, 24 h, 48 h, 5 d, and 10 d after surgery (Table 2).

#### 4.4.3. Hematology

On the 10th day after surgery, after animals were anesthetized under isoflurane, blood was collected via the intracardiac route to determine the total red blood cells, hemoglobin, hematocrit, secondary red blood indices, total white blood cells, and total platelet counts.

#### 4.4.4. Infarct Size and Brain Edema Determination

Brain samples were cut cross-sectionally 2 mm after the level of the bregma. They were covered entirely with the staining solution, which is a mixture of 15 mL of 2% triphenyl tetrazolium chloride (TTC) and 1 mL of 1 M potassium phosphate (dibasic) diluted to 50 mL. The slices were incubated in the dark at 37 °C for 30 min and turned once after 15 min to promote an even reaction. Normal tissue produces a brick-red reaction product with the reduction of TTC by intracellular dehydrogenases, whereas ischemic tissue remains unreacted and has uncolored or pale staining. Once exposed to TTC, the brain slices were photographed using a camera, and the infarct size was measured by computing the infarcted area using Adobe^®^ Photoshop CS6 [40]. The background of the pictures was changed to black, and each section was assessed by dividing the number of pixels representing pale staining areas by the total area of the brain section. The infarcted area is computed using the following formula:

$$Infarct\;Size=\frac{Infarcted\;area}{Total\;Brain\;area}\times 100$$

The area of brain edema for both ipsilateral (injured) and contralateral (uninjured) hemispheres was manually traced, and the number of pixels was computed as discussed for infarct size determination [40]. Ipsilateral hemispheric swelling was calculated using the following formula:

$$Ipsilateral\;Hemispheric\;Edema=\frac{Ipsilateral\;area-Contralateral\;area}{Contralateral\;area}\times 100$$

#### 4.4.5. Histopathological Analysis of Brain Tissues

The collected brains were processed for routine histopathological analysis using H and E staining. In brief, the brain samples were cut cross-sectionally at the bregma level, and representative samples were examined for hypoxic neuronal cells. The number of hypoxic neuronal cells was counted in ten random fields (200×), averaged per animal, and reported as a percentage (%). Other notable features, such as leukocyte infiltration and cerebral and subarachnoid hemorrhages, were likewise noted.

#### 4.4.6. Statistical Analysis for Behavioral and Morphophysiological Studies

The *t*-test was used for the different hematological parameters, infarct size, ipsilateral hemispheric edema, and hypoxic neurons, which are reported as mean ± SD. Two-way analysis of variance (ANOVA) with Bonferroni’s test for multiple comparison was used to compare the neurologic score, which is reported as mean ± SD. Statistical significance was determined at *p* < 0.05.

### 4.5. Gene Expression Studies

#### 4.5.1. Animal Experimental Procedures for Gene Expression Studies

SD rats were randomly assigned into two groups with 25 animals each: Tween-80 and VCO, both treatments given orally at 5 mL/kg/day. After one week of acclimatization, five rats from each group were sacrificed to determine baseline parameters. After which, the treatments are administered. After five days of treatment, five rats were again sacrificed to determine whether VCO may have affected the parameters to be observed. By this time, the remaining rats were then subjected to left extracranial internal carotid artery occlusion (ECICAO) before administering the dose for the 6th day. The procedure performed followed what has been discussed in Diestro, Omar, Climacosa, Mondia, Arbis, Collantes, Khu, Roxas, and Estacio [11], except that the occlusion was permanent in this model under isoflurane anesthesia. Five animals were then sacrificed each at 4 h, 24 h, and 48 h after the surgery.

#### 4.5.2. Preparation of Brain Tissue

Animals were euthanized by anesthetizing the animals with isoflurane before having the head decapitated and the brain removed within 3 min. The brain samples were dissected, and the left cerebral cortex was stored at −80 °C until the time total RNA isolation was performed.

#### 4.5.3. Measurement of Relative Gene Expression Levels

RNA Isolation. Total RNA was isolated in TRIzol following following manufacturers instructions. In general, the brain sample was homogenized in 1 mL of TRIzol reagent per 50 mg of sample (ThermoFisher Scientific, Walthan, MA, USA) and a cell homogenizer (Shake Master Auto; Hirata Corporation, Kumamoto, Japan) for 3 min. The RNA concentration was determined through spectrophotometric analysis (NanoDrop One; ThermoFisher Scientific, Walthan, MA, USA).

Complementary DNA (cDNA) Synthesis. RT-PCR was performed using ReverTRA ACE^TM^ qPCR RT Master Mix with gDNA Remove (Toyobo, Osaka, Japan) following manufacturer’s instructions.

Quantitative Real-Time Polymerase Chain Reaction (qPCR). qPCR was conducted for caspase-3 (*Cas 3*), receptor for advanced glycation end products (*Rage*), angiogenin (*Ang*), angiopoietin 1 (*Angpt 1*), dynamin-related protein 1 (*Drp 1*), mitofusin 2 (*Mfn 2*), parkin, mitochondrial rho (*Miro 1*), and glyceraldehyde-3-phosphate dehydrogenase (*Gapdh*). Hypoxanthine phosphoribosyl transferase 1 (*Hprt 1*) was used as an internal control, as compared with *Gapdh* because *Hprt 1* has a more relatively consistent expression levels in stroke-induced models. The primers were obtained from FASMAC (Kanagawa, Japan). The primer sequences are as follows:

Cas 3 Forward: GTG GAA CTG ACG ATG ATA TGG C

Cas 3 Reverse: CGC AAA GTG ACT GGA TGA ACC

Rage Forward: CCC TGA CCT GTG CCA TCT CT

Rage Reverse: GGG TGT GCC ATC TTT TAT CCA

Ang Forward: CAC AGA TGG CCT TGA TGT TG

Ang Reverse: CTC TGG CTC AGC ATG ACT CC

Angpt 1 Forward: GCC ACT TGA GAA TTA CAT TGT GG

Angpt 1 Reverse: CGC GGA TTT TAT GCT CTA ATC AAC TG

Drp 1 Forward: GCA GCC GTA GTC CTC AAA GA

Drp 1 Reverse: CTC CAC CTT TTG AAG CCA GG

Mfn 2 Forward: AGT CGG TTG GAA GTC ACT GT

Mfn 2 Reverse: TGT ACT CGG GCT GAA AGG AG

Parkin Forward: CTG GCA GTC ATT CTG GAC AC

Parkin Reverse: CTC TCC ACT CAT CCG GTT TG

Gapdh Forward: TCA CCA CCA TGG AGA AGG

Gapdh Reverse: GCT AAG CAG TTG GTG GTG CA

Hprt 1 Forward: TGT TTG TGT CAT CAG CGA AAG TG

Hprt 1 Reverse: ATT CAA CTT GCC GCT GTC TTT TA

Ultrapure water was produced with the Milli-Q ultrapure water system (Millipore, Bedford, MA, USA). The ultrapure water was added to each cDNA sample to obtain a 10× dilution for qPCR. The standard curve was made by using duplicates of 10×, 40×, 160×, 640×, and 2560× of a sample’s cDNA. The ultrapure water was used as a negative control by replacing the cDNA sample in the reaction mixture. The composition of 10× diluted cDNA, 0.5 mM primer, and THUNDERBIRD^TM^ SYBR^TM^ qPCR Mix (Toyobo, Osaka, Japan) (Table 3).

The qPCR amplification was performed on an Applied Biosystems^TM^ StepOnePlus^TM^ Real-Time PCR System (ThermoFisher Scientific, Waltham, MA, USA) with the following conditions:Pre-denaturation95 °C30 s
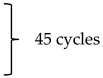
Denaturation95 °C10 sAnnealing58 °C10 sExtension72 °C12 s

The sample’s mRNA levels were normalized using *Hprt 1* mRNA for the quantitative evaluation of gene expression.

#### 4.5.4. Statistical Analysis for Gene Expression Studies

Two-way ANOVA with Bonferroni’s test for multiple comparisons was used to compare the relative gene expression, which is reported as mean ± SD. Statistical significance was determined at *p* < 0.05.

## Figures and Tables

**Table 1:** Fatty acid profile of virgin coconut oil obtained from a local supermarket in the Philippines.

Carbon Length.	Component	%
C6	Caproic Acid	0.3154 ± 0.0028
C8	Caprylic Acid	5.9243 ± 0.0108
C10	Capric Acid	4.8752 ± 0.0574
C12	Lauric Acid	52.3606 ± 0.0145
C14	Myristic Acid	19.0247 ± 0.0102
C16	Palmitic Acid	8.5672 ± 0.0293
C17	Stearic Acid	2.2450 ± 0.0132
C18	Oleic Acid	5.7862 ± 0.0204
C18:1n9c	Linoleic Acid	0.9013 ± 0.0364

**Table 2:** Neurological scoring used to determine whether the individuals had experienced stroke or not [9].

Score *	Description
0	No neurological deficit
1	Left forelimb flexion when suspended by the tail or failure to extend the right forepaw fully
2	Left shoulder adduction when suspended by the tail
3	Reduced resistance to lateral push toward the left side
4	Spontaneous movements in all directions with circling to the left exhibited only if pulled by the tail
5	Circle or walk spontaneously only to the left.
6	Walk only when stimulated.
7	No response to stimulation
8	Stroke-related death

* Rats were identified to have experienced stroke if they were classified as having a score of 1 and above.

**Table 3:** Preparation of Samples for qPCR Amplication.

10× diluted cDNA	2 μL
ultrapure water	6.6 μL
forward primer	0.5 μL
reverse primer	0.5 μL
THUNDERBIRD^TM^ SYBR^TM^ qPCR Mix	10 μL
ROX Dye	0.4 μL
Total Volume	20 μL

## Data Availability

The raw data supporting the conclusions of this manuscript will be made available by the authors, without undue reservation, to any qualified researcher.

## Abbreviations

The following abbreviations are used in this manuscript:

*Ang*	angiogenin
*Angpt 1*	angiopoietin 1
ANOVA	analysis of variance
BP	blood pressure
cDNA	complementary deoxyribonucleic acid
*Drp 1*	dynamin-related protein 1
ECICAO	extracranial internal carotid artery occlusion
*Gapdh*	glyceraldehyde-3-phosphate dehydrogenase
*Hprt 1*	hypoxanthine phosphoribosyl transferase 1
HPLC	high-performance liquid chromatography
HR	heart rate
MCH	mean corpuscular hemoglobin
MCHC	mean corpuscular hemoglobin concentration
MCV	mean corpuscular volume
*Mfn 2*	mitofusin 2
*Miro*	mitochondrial rho
qPCR	Quantitative real-time polymerase chain reaction
*Rage*	receptor for advanced glycation end-products
RBC	red blood cells
RNA	ribonucleic acid
ROS	reactive oxygen species
SD	Sprague Dawley
SHRSP	stroke-prone spontaneously hypertensive rat
TTC	triphenyl tetrazolium chloride
VCO	virgin coconut oil
WBC	white blood cells
